# CyberKnife Staged Radiotherapy for Elderly Patients With Poorly Differentiated Lung Adenocarcinoma: A Case Report and Dosimetric Analysis

**DOI:** 10.7759/cureus.83378

**Published:** 2025-05-02

**Authors:** Shanshan Gu, Xiaoliang Liu, Lehui Du, Zhongjian Ju, Haiyang Wang, Jinglin Sun, Xiangkun Dai

**Affiliations:** 1 Medical Physics, Department of Radiation Oncology, The First Medical Center of PLA General Hospital, Beijing, CHN; 2 Radiation Oncology, Department of Radiation Oncology, The First Medical Center of PLA General Hospital, Beijing, CHN; 3 Radiodiagnosis, Department of Radiation Oncology, The First Medical Center of PLA General Hospital, Beijing, CHN

**Keywords:** copd, cyberknife, elderly patients, offline adaptive radiotherapy, poorly differentiated lung adenocarcinoma

## Abstract

This case describes an 81-year-old male patient with poorly differentiated lung adenocarcinoma, complicated by severe chronic obstructive pulmonary disease (COPD) and a history of coronary stenting. The patient underwent CyberKnife (Accuray Inc., Madison, WI, United States) stereotactic body radiotherapy (SBRT) with synchrony respiratory tracking and staged radiotherapy. The patient received staged radiotherapy delivered in two sequential phases: phase I (30 Gy in three fractions) was administered from November 18 to 24, 2022, followed by phase II (30 Gy in three fractions) initiated on February 20, 2023, after a 12-week intermission for therapeutic response assessment and normal tissue recovery. The cumulative prescription totaled 60 Gy in six fractions (equivalent dose in 2-Gy fractions (EQD2) = 120 Gy, α/β = 10), with strict adherence to organ-at-risk (OAR) constraints. Through fiducial marker implantation and dynamic dose optimization, the ipsilateral lung V20 was reduced from 3.35% to 1.63%. No severe treatment-related toxicities (≥ grade 2) were observed during or after the treatment period. At a three-month follow-up, the tumor volume decreased by 79% (117.04 cm³ → 24.26 cm³), demonstrating significant local control. This case provides a practical reference for personalized radiotherapy in elderly patients with cardiopulmonary comorbidities.

## Introduction

Lung cancer remains the leading cause of cancer-related mortality worldwide, with over 1.8 million annual deaths reported in 2020, of which non-small cell lung cancer (NSCLC) accounts for approximately 85% of cases [1]. Among early-stage NSCLC patients, nearly 30% are deemed medically inoperable due to advanced age (≥75 years) or cardiopulmonary comorbidities [2]. Stereotactic body radiotherapy (SBRT) has been established as the standard-of-care for this population, delivering submillimeter targeting accuracy and abbreviated treatment courses (typically 3-5 fractions over 1-2 weeks) [3]. However, respiratory-induced tumor motion, particularly for lesions greater than 5 cm in diameter, poses a significant challenge in balancing target coverage and organ-at-risk (OAR) sparing (e.g., the lungs and the heart) using conventional fixed-field techniques [4].

Respiratory excursions may induce tumor displacement amplitudes of 2-3 cm, necessitating larger planning target volume (PTV) margins (5-10 mm in conventional radiotherapy), which consequently increases normal lung irradiation (e.g., bilateral lung V20 > 15%) [5]. The CyberKnife System (Accuray Inc., Madison, WI, United States) achieves dynamic tumor tracking through multimodal correlation modeling between intratumoral fiducial markers and external respiratory motion monitoring [6]. During treatment delivery, dual orthogonal X-ray imaging devices continuously capture the positional coordinates of implanted fiducials at a sampling rate of 30 Hz, while infrared cameras monitor the displacement of surface markers on the thoracoabdominal region. These datasets are integrated into the Synchrony® respiratory correlation model (Accuray Inc., Madison, WI, United States) to predict three-dimensional tumor trajectories in real time, accounting for respiratory-induced motion. When deviations in respiratory patterns occur (e.g., irregular breathing or cough-induced motion), the system dynamically adjusts the beam orientation via a robotic manipulator arm with submillimeter accuracy (root-mean-square error < 0.5 mm), ensuring continuous radiation focus on the tumor centroid. This method enables PTV margin reduction to 2-3 mm while maintaining target coverage [7]. For bulky tumors (>5 cm), non-coplanar beam arrangements combined with spherical dose convergence significantly reduce high-dose exposure to adjacent critical structures [8]. Clinical studies demonstrate that this approach achieved remarkable dual efficacy, with bystander and abscopal response rates reaching 96% and 52%, respectively. Tumor burden analysis revealed a median shrinkage of 70% (range 30%-100%) in partially irradiated bulky tumors demonstrating bystander effects, while non-irradiated metastases exhibiting abscopal effects showed comparable median reduction of 50% (range 30%-100%). Importantly, the treatment maintained an exemplary safety profile, with no patients experiencing acute or late toxicity of any grade [9]. However, the treatment of bulky tumors remains challenging due to steep dose fall-off gradients and insufficient coverage at target margins, necessitating further optimization of respiratory gating algorithms and dose distribution models [10].

The CyberKnife system employs image-guided tracking and a six-dimensional robotic arm to enable real-time correction of tumor displacement during treatment, achieving submillimeter accuracy (<1 mm) that surpasses that of conventional radiotherapy systems [11]. This technology is particularly advantageous for lower-lobe tumors exhibiting diaphragmatic-driven displacements of 2-3 cm [12]. For patients with complex respiratory patterns (e.g., chronic obstructive pulmonary disease, COPD), staged radiotherapy improves dose optimization through sequential plan re-optimization based on anatomical changes observed during treatment [13]. This reduction in lung dose may mitigate radiation-induced pulmonary toxicity, particularly in patients with pre-existing functional compromise.

This case illustrates the successful application of CyberKnife with Synchrony and staged radiotherapy in an 81-year-old patient with cardiopulmonary dysfunction and targetable mutation-negative NSCLC, achieving tumor control and OAR sparing. The findings provide practical insights for managing complex cases where conventional therapeutic options are limited.

## Case presentation

In June 2021, an 81-year-old male patient presented with an incidental pulmonary mass (22 × 12 mm) in the left lower lobe, detected during routine screening. PET-CT demonstrated a hypermetabolic nodule (SUVmax 8.7), suggestive of malignancy. Pathological confirmation via CT-guided biopsy revealed poorly differentiated adenocarcinoma, with immunohistochemistry showing focal CK7-positive and a Ki-67 proliferation index of 70%. Genetic testing (EGFR/ALK/ROS1) was negative.

**Figure 1:**
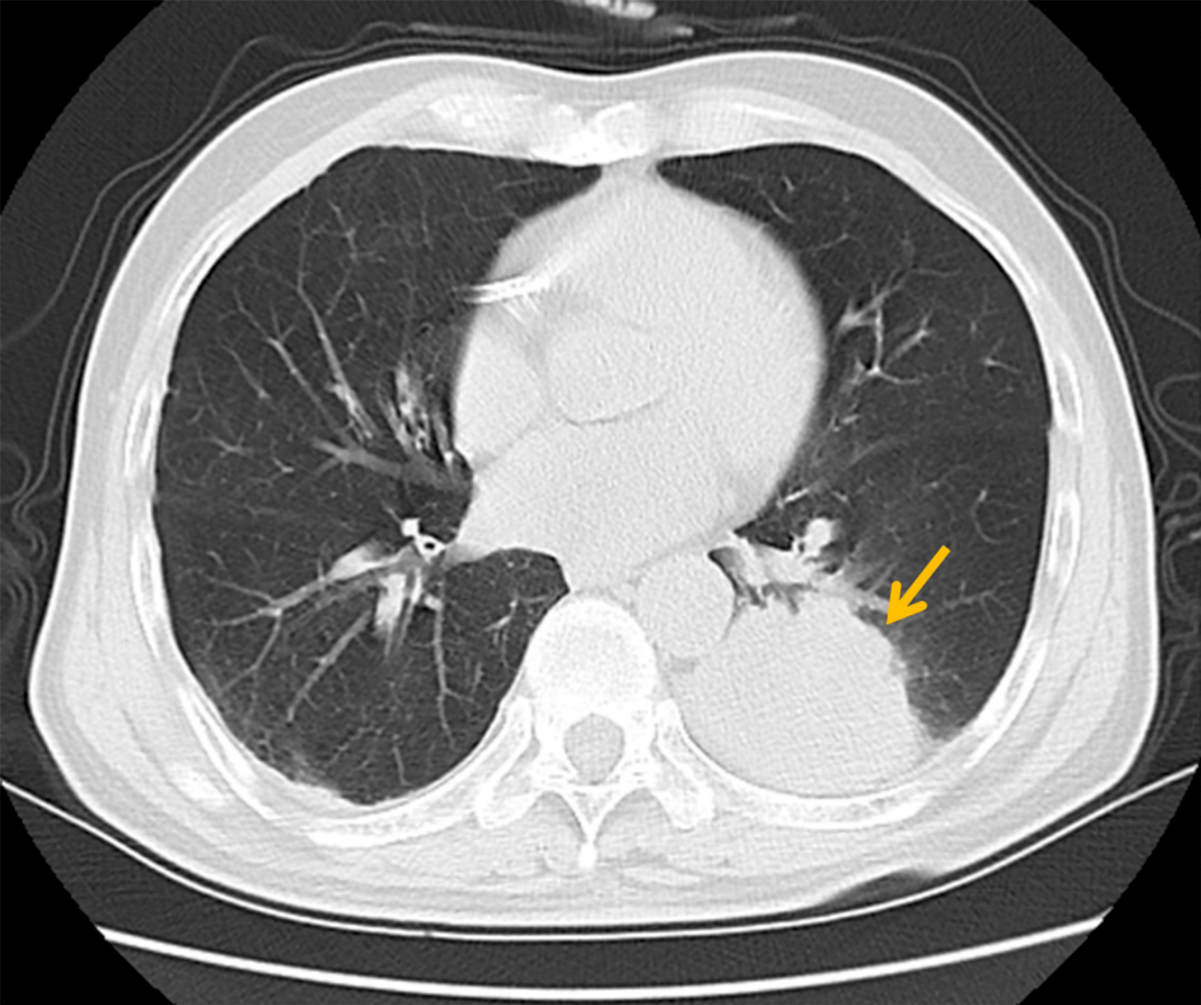
Chest CT showing a 6-cm mass in the left lower lobe (arrow)

The patient had an eight-year history of severe COPD (FEV1/FVC 48%, severe mixed ventilatory dysfunction) and a 14-year status post-coronary stent implantation. Deemed inoperable by multidisciplinary thoracic surgery evaluation, the patient declined systemic chemotherapy and conventional fractionated radiotherapy (typically 15-35 fractions over 3-7 weeks). The refusal stemmed from concerns regarding physical tolerance of extended treatment courses, consistent with documented challenges in treatment completion among frail patients [14]. Serial imaging modalities revealed progressive disease. A PET-CT performed in August 2021 showed tumor enlargement to 53 × 50 mm, and a chest CT in October 2022 confirmed further progression to 6 cm, with staging of cT3N0M0 (Figure 1).

**Figure 2:**
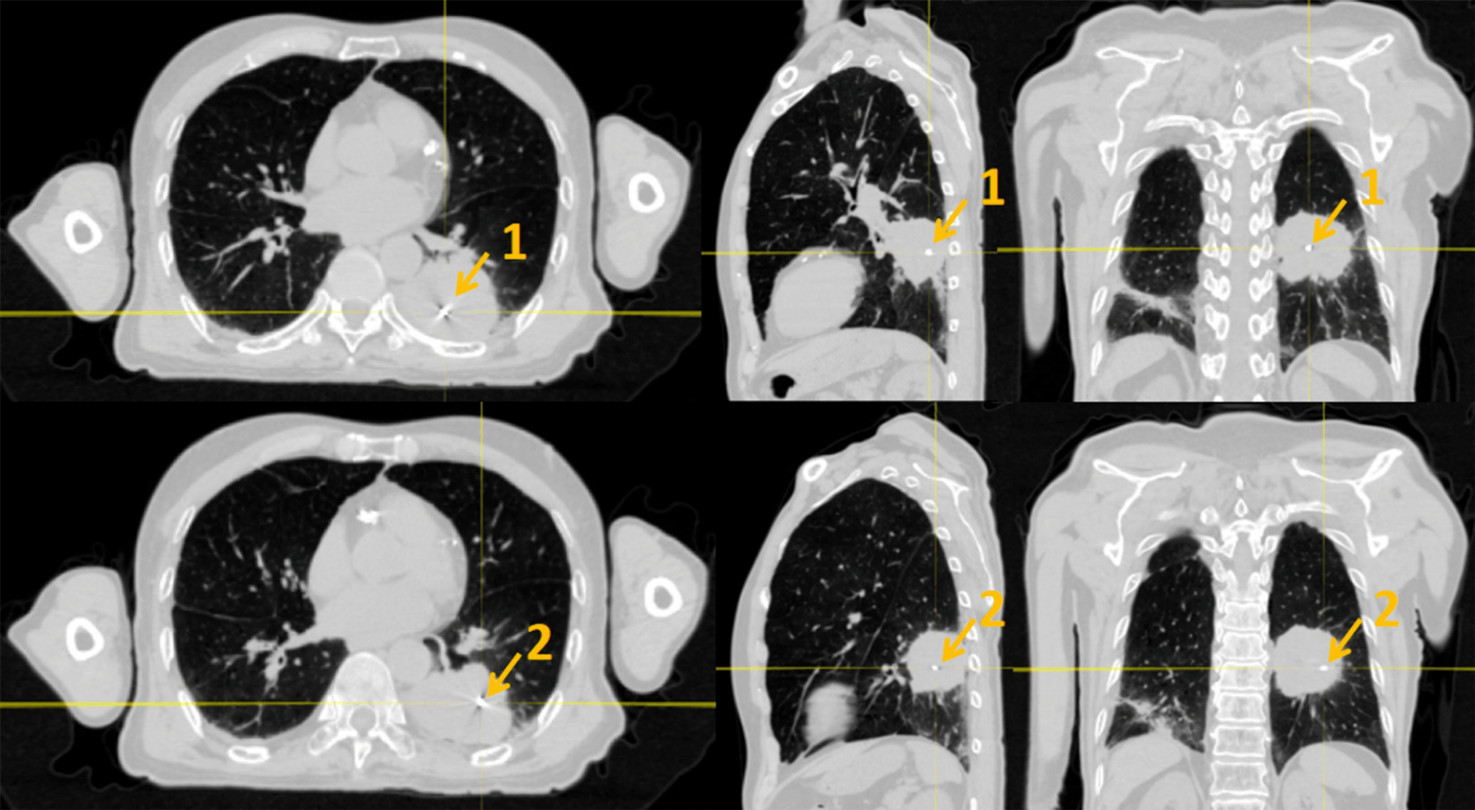
Schematic of fiducial marker placement (arrows) The gold marker is a small bright spot with a dense mass and radiographic artifacts: (1) first gold marker and (2) second gold marker (from left to right: cross-sectional, coronal, sagittal plane).

Owing to cardiac shadowing obscuring the tumor on orthogonal 45° kV projections, a recognized limitation of pulmonary surrogate tracking, fiducial marker-based respiratory motion tracking was selected. This decision aligned with our institutional experience, demonstrating that fiducial implantation provides enhanced visualization and more accurate representation of true tumor kinematics in the presence of cardiopulmonary anatomical interference. Two gold fiducial markers were percutaneously implanted under CT guidance within the lesion periphery (Figure 2). A correlation model between the external surface motion (tracked via infrared markers) and internal fiducial displacement was established, achieving intrafractional tracking accuracy below 1 mm during treatment sessions. This motion-compensation strategy enabled precise targeting while sparing functional lung parenchyma.

Staged radiotherapy protocol

The therapeutic approach comprised two sequentially optimized SBRT phases using CyberKnife. Phase I (November 18, 2022) delivered 30 Gy in three fractions (BED = 60 Gy, α/β = 10) to the gross tumor volume (GTV), intentionally excluding subclinical margins to achieve rapid tumor volume reduction while minimizing pulmonary radiation exposure. Following the interval imaging demonstrating partial response, the second treatment phase (February 20, 2023) delivered a 30-Gy boost dose in three fractions (BED = 60 Gy, α/β = 10) to the PTV. This PTV was defined by applying a 5-mm isotropic margin to the post-phase I reduced GTV, accounting for (1) potential microscopic disease extension beyond the CT-visible residual tumor and (2) inherent setup uncertainties associated with multiphase SBRT delivery, consistent with staged dose escalation protocols for hypofractionated regimens. Staged radiotherapy achieved ipsilateral lung V20 reduction (3.35% → 1.63%) through interphase dose optimization, reflecting refined dosimetric sparing of pulmonary tissue.

Imaging and treatment planning

**Figure 3:**
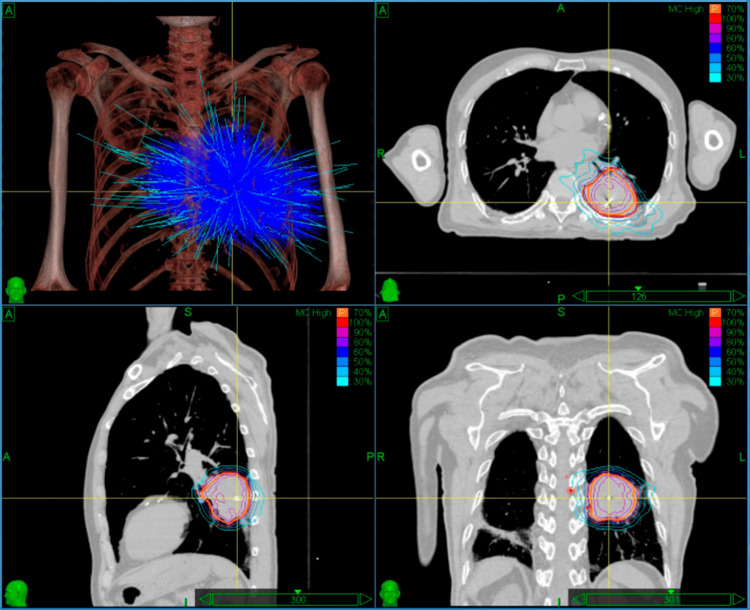
Dose distribution of the initial treatment plan (prescription dose: 30 Gy in three fractions) Within the treatment planning system interface for the first fraction, the dose cloud is displayed using normalized dose scaling, with the outermost isodose line representing 30% of the maximum dose (Dmax).

**Figure 4:**
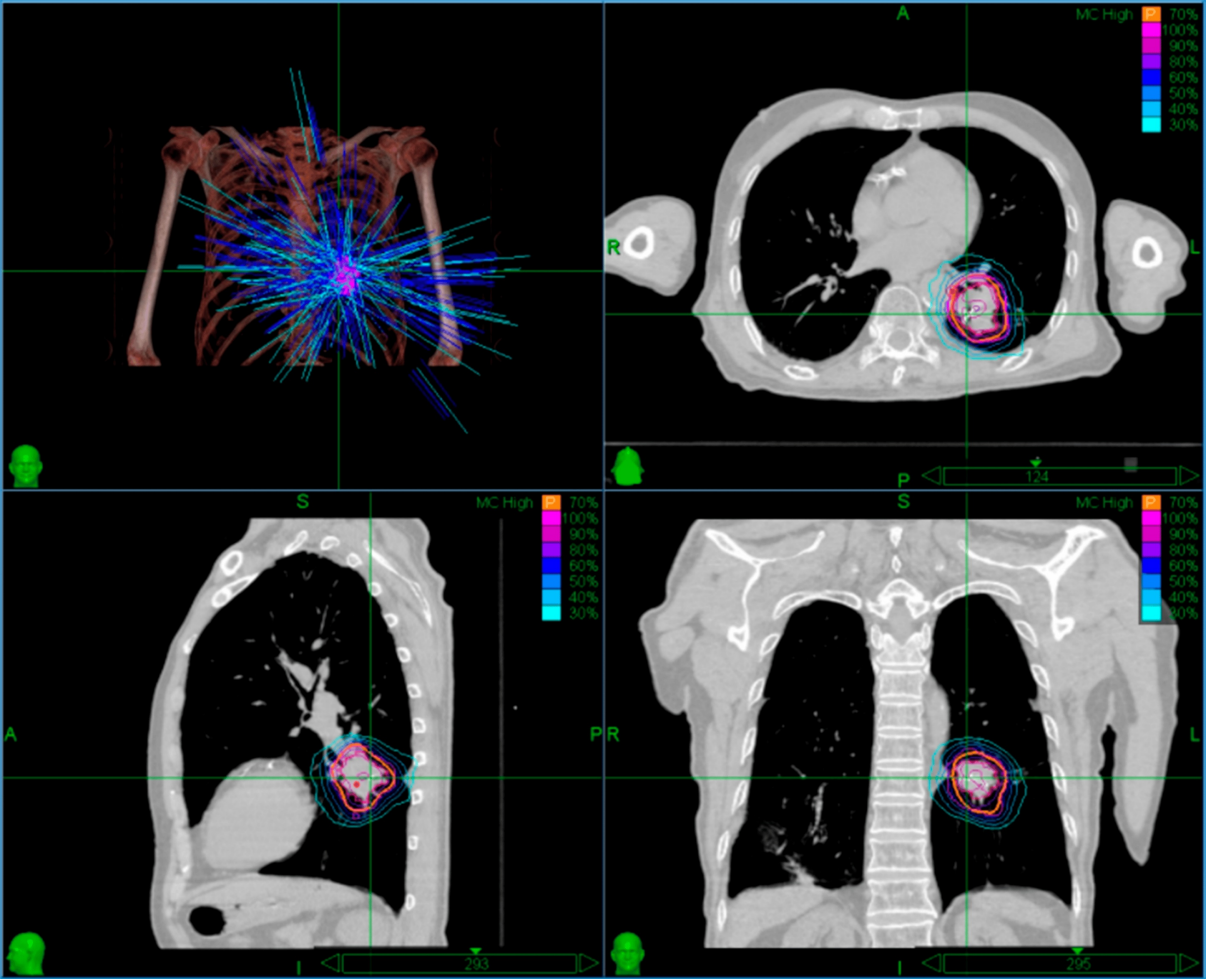
Three-dimensional dose distribution of the staged radiotherapy replanning strategy (prescription dose: 30 Gy in three fractions) Within the treatment planning system interface for the second fraction, the dose cloud is displayed using normalized dose scaling, with the outermost isodose line representing 30% of the maximum dose.

Pretreatment simulation utilized a vacuum cushion immobilization system with supine positioning. High-resolution planning images were acquired via 1-mm slice thickness scanning on a Siemens SOMATOM Definition AS large-bore CT scanner (Siemens Healthineers, Erlangen, Germany). The initial treatment plan utilized 15-mm and 35-mm collimators selected based on the target maximum diameter of 60 mm, employing 64 nodes with a total monitor unit (MU) count of 44,913.2. Through 147 non-coplanar beams, the plan achieved 68.2% GTV coverage (GTV 117 cm³) and delivered a maximum central dose of 42.9 Gy (Figure 3). For re-planning in fractionated radiotherapy, a repeat CT simulation was performed to assess tumor regression. The secondary radiotherapy plan optimized target coverage to 73.6% for the PTV (58.1 cm³) and 99.7% for the residual GTV (24.3 cm³) using 20-mm and 30-mm collimators selected based on the target maximum diameter of 41 mm, employing 47 nodes with a total MU count of 30,017.8 across 111 beams. This configuration maintained equivalent peak dose constraints while reducing treatment time from 77 to 61 minutes (Figure 4).

Dose constraints for OARs were established in compliance with the AAPM Task Group 101 Report [15]. OAR dose metrics for both treatment plans are presented in Table 1.

**Table 1:** Dose metrics of organs at risk for two treatment plans

OAR	Dose type	Volume (cc/%)	Dose (first fraction)	Dose (second fraction)
Esophagus	Dmax (Gy)		13.8	8.4
	Volume (cc)	<5	13.6	8.1
Trachea	Dmax (Gy)		23.5	8.3
	Volume (cc)	<4	23.2	8.1
Heart	Dmax (Gy)		17.8	13.5
	Volume (cc)	<15	17.5	13.1
Aorta	Dmax (Gy)		30.1	28.4
	Volume (cc)	<10	29.8	25.7
Chest wall	Dmax (Gy)		23	18.6
	Volume (cc)	<1	22.8	18.4
Lung-L	v20 Gy (%)		2.8	1.3

Both plans utilized real-time respiratory tracking, and patient setup time was estimated at 15 minutes, with imaging verification intervals of 45 seconds during treatment. During the delivery of two radiotherapy plans, beam activation in the CyberKnife system adhered to six-dimensional spatial accuracy criteria, requiring rotational errors (Pitch, Yaw, Roll) to remain below 1° and translational errors (X, Y, Z) to be less than 1 mm. Rotational deviations were monitored using spinal tracking-assisted planning, while translational displacements were tracked via implanted fiducial markers. Throughout the treatment, real-time image-guidance systems continuously assessed target positioning through bony landmarks or fiducials. When deviations exceeded predefined thresholds, the six-dimensional robotic arm dynamically corrected errors by compensating for translational shifts and adjusting rotational misalignments, ensuring submillimeter accuracy for the target volume. This precision maintained optimal tumor coverage while significantly reducing the risk of normal tissue complications, aligning with clinical standards for stereotactic radiotherapy.

After detailed discussions regarding indications, procedures, and potential side effects, the patient provided verbal and written informed consent.

Follow-up and outcomes

**Figure 5:**
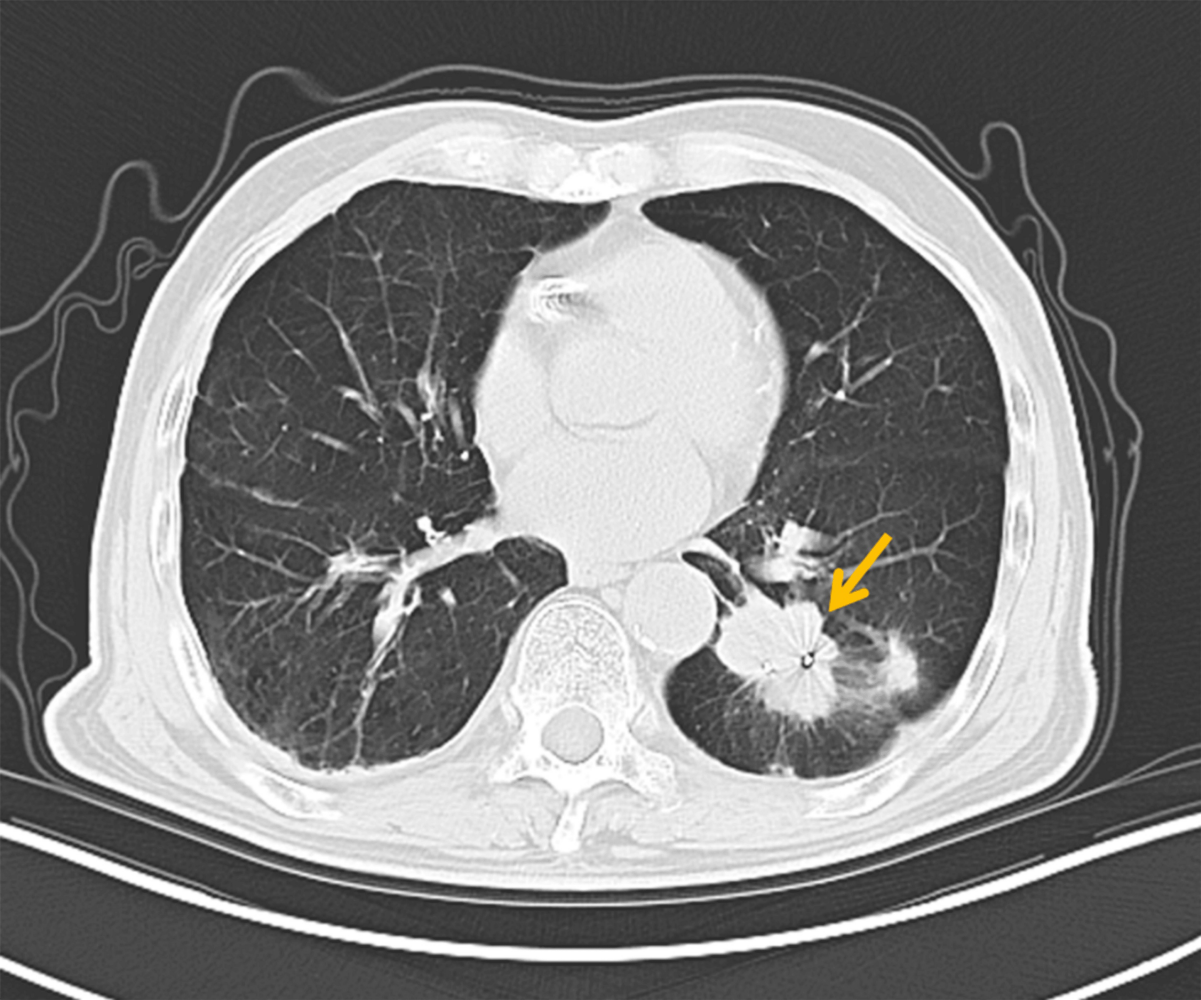
Follow-up imaging three months after initial treatment showing tumor reduction to 43 × 30 mm (arrow)

**Figure 6:**
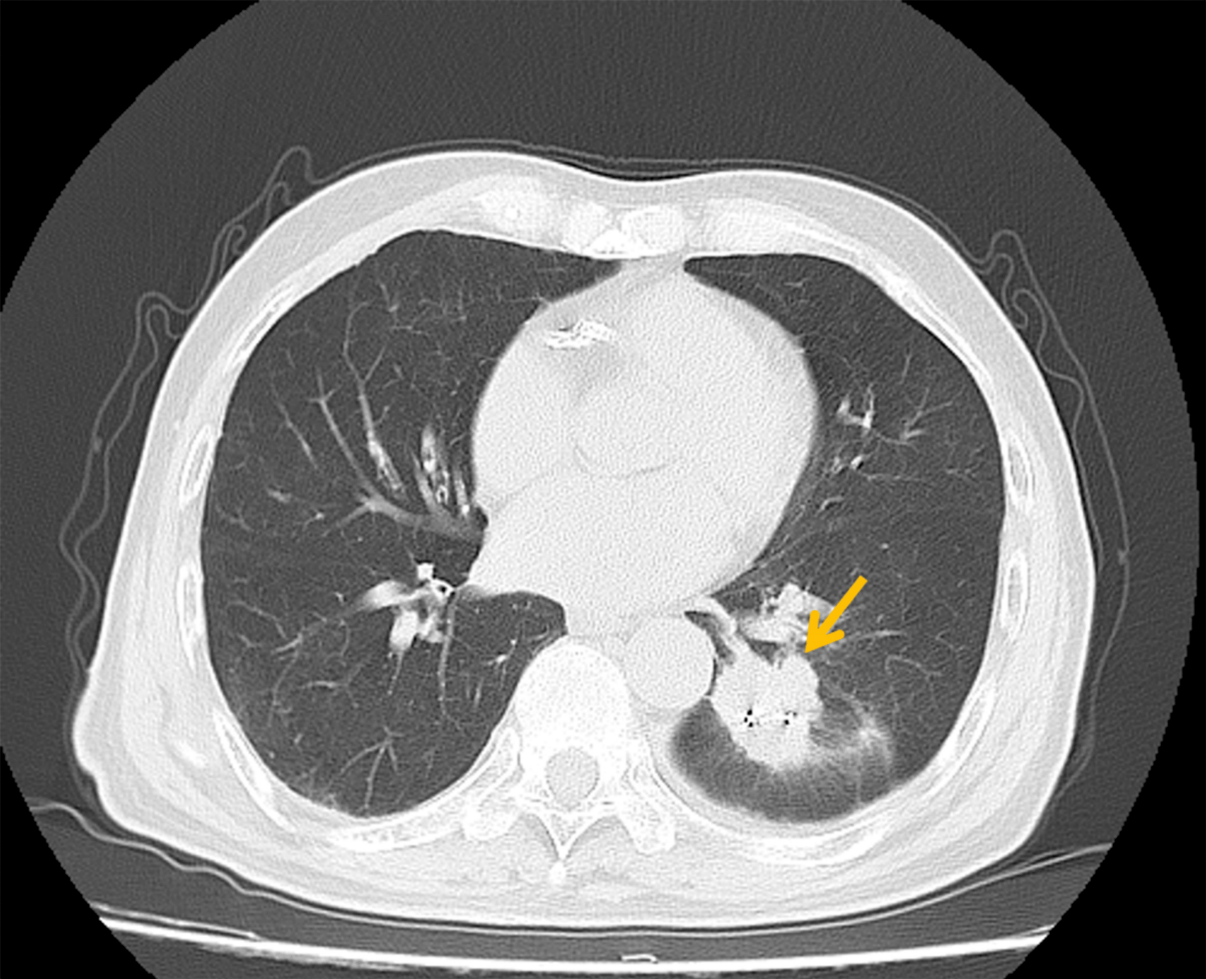
Follow-up imaging six months after initial treatment showing tumor size of 41 × 32 mm (arrow), prompting initiation of the second treatment phase

**Figure 7:**
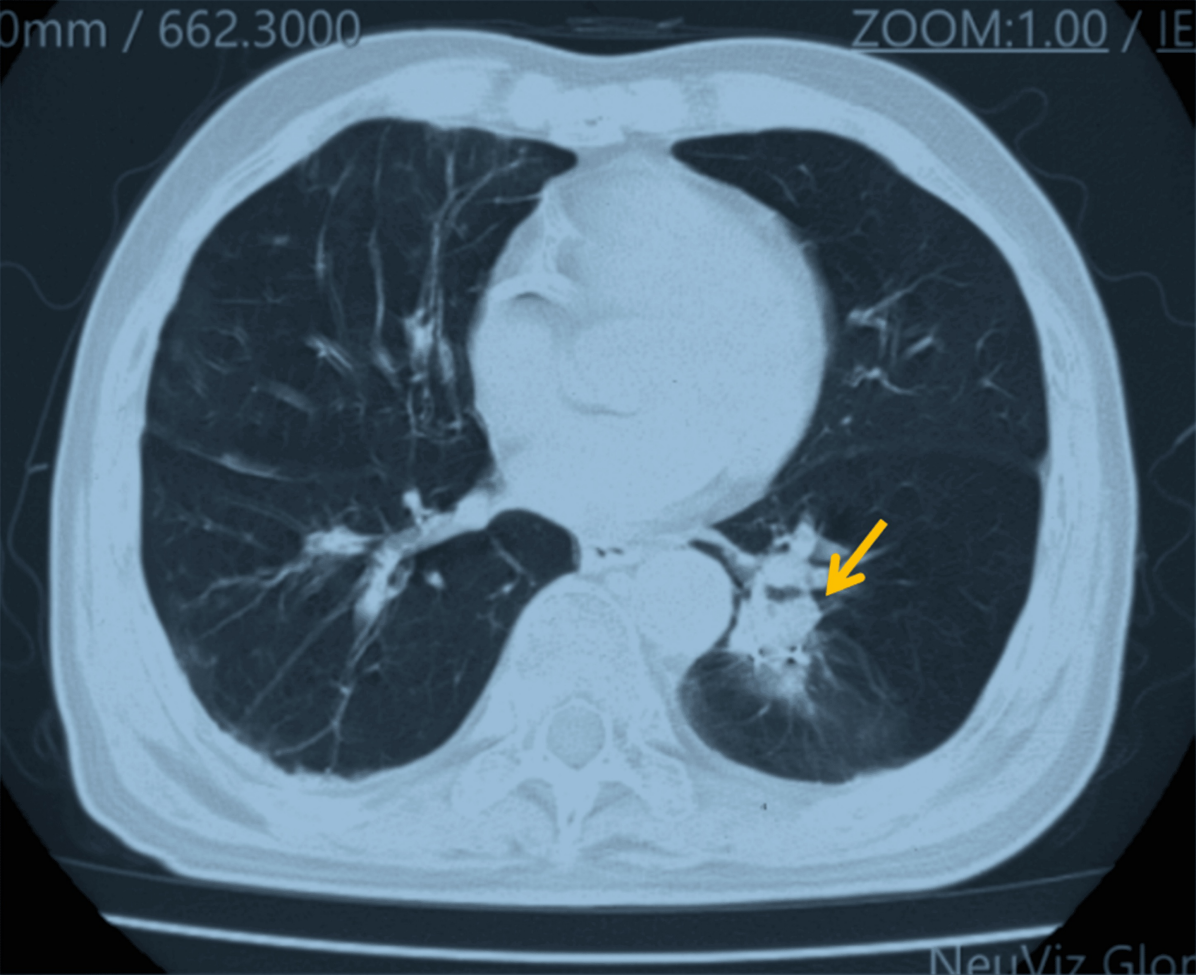
Follow-up imaging six months after re-treatment showing tumor reduction to 20 × 20 mm (arrow)

Serial imaging surveillance demonstrated a sustained treatment response. A chest CT in December 2022 revealed tumor reduction to 43 × 30 mm (Figure 5), with further regression to 41 × 32 mm by February 2023 (Figure 6), prompting phase II irradiation. Six months post-retreatment (September 2023), the lesion measured 20 × 20 mm (Figure 7).

Safety

No grade ≥2 radiation pneumonitis (per CTCAE v5.0), pericarditis, or significant arrhythmias were observed, consistent with the low-toxicity profile of SBRT [16]. Dynamic pulmonary function tests revealed stable FEV1 at 0.98 L (82% predicted), with <5% fluctuation from baseline, indicating no progressive pulmonary impairment [17]. No delayed complications (e.g., fibrosis and bronchial stenosis) occurred during follow-up. The Karnofsky Performance Status (KPS) remained stable at 90, and the EORTC QLQ-C30 global quality-of-life score improved by 8% from baseline [18,19]. As of the last follow-up (March 2024), progression-free survival reached 18 months, with an ECOG performance status of 0 and no late-term complications. Overall survival (OS) remains undetermined, with ongoing monitoring.

Ethical considerations

The patient provided written informed consent for both clinical procedures and anonymized publication of this case report, including de-identified medical images and clinical data. This study adhered to the HIPAA Safe Harbor criteria for data anonymization. All personal identifiers were removed from radiographic materials and clinical records in accordance with the ICMJE guidelines.

## Discussion

In elderly patients with stage T3 NSCLC and cardiopulmonary dysfunction, CyberKnife-based staged SBRT employing a two-phase dose optimization strategy effectively balances tumor control and normal tissue protection. For this 81-year-old patient (with COPD and a history of coronary stent implantation), surgical resection and conventional radiotherapy were contraindicated. A staged CyberKnife protocol was implemented: during the first stage (30 Gy/3 fractions), rapid tumor volume reduction (GTV from 117 cm³ to 58.1 cm³) minimized subsequent pulmonary irradiation. The second stage (30 Gy/3 fractions) utilized adaptive replanning with a 5-mm PTV expansion based on tumor regression, achieving a cumulative biologically effective dose (BED10) of 120 Gy. This approach reduced the ipsilateral lung V20 from 2.8% to 1.3%, significantly surpassing reported values for single-stage SBRT (mean V20: 5-8%) [16] and avoiding grade ≥2 radiation pneumonitis, thereby validating the lung-sparing advantage of staged radiotherapy.

The CyberKnife Synchrony respiratory tracking system overcame challenges posed by severe COPD and pulmonary embolism, including excessive respiratory motion (diaphragmatic excursion > 2 cm) and inability to perform breath-hold immobilization. By establishing real-time correlation modeling between implanted fiducial markers and external surrogates, it achieved dynamic correction of six-dimensional spatial errors (translational < 1 mm, rotational < 1°). Compared to conventional radiotherapy with fixed margins (typically 5-10 mm), the second-phase PTV expansion of 5 mm in this case reduced irradiated lung volume by approximately 30%, adhering to the precision requirements of AAPM Task Group 101 for stereotactic radiotherapy. A 12-week interphase allowed assessment of tumor biology and pulmonary functional recovery, a strategy proven to enhance tolerance in frail elderly patients [20]. Eighteen-month follow-up demonstrated a 79% tumor volume reduction (117.04 cm³ → 24.26 cm³), sustained KPS of 90, and absence of grade ≥2 toxicities, consistent with reported outcomes for elderly patients receiving SBRT with BED10 ≥ 100 Gy (three-year local control, 79.2%; grade ≥3 toxicity, <2.9%) [21].

However, patient selection for staged radiotherapy requires stringent criteria. Tumor biology (e.g., aggressive subtypes) may influence therapeutic efficacy, necessitating potential synergy with immunotherapy. Additionally, fiducial marker implantation carries the risk of pneumothorax, mandating comprehensive preoperative evaluation [22]. Multicenter randomized trials are imperative to validate long-term survival benefits and applicability in T3-stage NSCLC.

## Conclusions

This case demonstrates the feasibility of CyberKnife respiratory motion management in a high-risk elderly patient with pulmonary embolism who declined surgery and conventional radiotherapy. The two-stage dose strategy (total BED10 = 120 Gy) combined with real-time six-dimensional error correction (translation < 1 mm, rotation < 1°) achieved remarkable tumor reduction (GTV 117.04 cm³ → 24.26 cm³) without grade ≥2 toxicities, aligning with established SBRT safety profiles. This paradigm offers personalized management for elderly patients with cardiopulmonary comorbidities, highlighting technical innovations (dynamic respiratory tracking, precision dose delivery) in complex cases. Further exploration of synergistic effects between staged radiotherapy and immunotherapy is warranted to optimize local control and survival outcomes.
